# Adhesion of rhomboid lip to lower cranial nerves as special consideration in microvascular decompression for hemifacial spasm: Report of two cases

**DOI:** 10.4103/2152-7806.72581

**Published:** 2010-11-18

**Authors:** Takeshi Funaki, Toshio Matsushima, Jun Masuoka, Yukiko Nakahara, Yukinori Takase, Masatou Kawashima

**Affiliations:** Department of Neurosurgery, Faculty of Medicine, Saga University, 5-5-1 Nabeshima, Saga, 849-8501, Japan

**Keywords:** Hemifacial spasm, infrafloccular approach, microvascular decompression, rhomboid lip

## Abstract

**Background:**

Although the rhomboid lip is a well-known structure constructing the foramen of Luschka, less attention has been directed to the structure for posterior fossa microsurgeries. The authors report two cases of the hemifacial spasm (HFS) with a large rhomboid lip, focusing on the importance of the structure during microvascular decompression.

**Case Description:**

A 59-year-old female presenting with left HFS was admitted to our hospital. A preoperative magnetic resonance image demonstrated an offending artery at the root exit zone of the VII nerve. The patient underwent microvascular decompression through the lateral suboccipital approach. The intraoperative findings showed that a large rhomboid lip adhered to the IX and X cranial nerves and prevented the exposure of the root exit zone of the VII cranial nerve. The rhomboid lip was meticulously separated from the cranial nerves so that the choroid plexus of the foramen of Luschka and the rhomboid lip could be safely lifted with a spatula, and the offending artery was successfully detached from the root exit zone. In another case of a 60-year-old male, the rhomboid lip was so large that it needed to be incised before separating it from the lower cranial nerves. In each case, the HFS was resolved following surgery without any new deficits.

**Conclusion:**

The large rhomboid lip adhering to the cranial nerves should be given more attention in the posterior fossa surgeries and should be managed based on the microsurgical anatomy for preventing unexpected lower cranial nerve deficit.

## INTRODUCTION

The choroid plexus protruding from the foramen of Luschka is an important landmark of the root exit zone of the VII cranial nerve during microvascular decompression surgery for hemifacial spasm (HFS).[1,4,5,10,12,13] The rhomboid lip, a sheet-like layer of neural tissue constructing the ventral wall of each lateral recess, is another structure that forms part of the foramen of Luschka.[3,6,11] Although the rhomboid lip is also a well-known structure in the field of microsurgical anatomy, less attention has been directed to the structure for the posterior fossa microsurgery. We report two cases in which a large rhomboid lip adhered to the IX and X cranial nerves during microvascular decompression for HFS, and discuss the operative management for such a situation based on the microsurgical anatomy.

## CASE REPORTS

### Case 1

**Figure 1:**
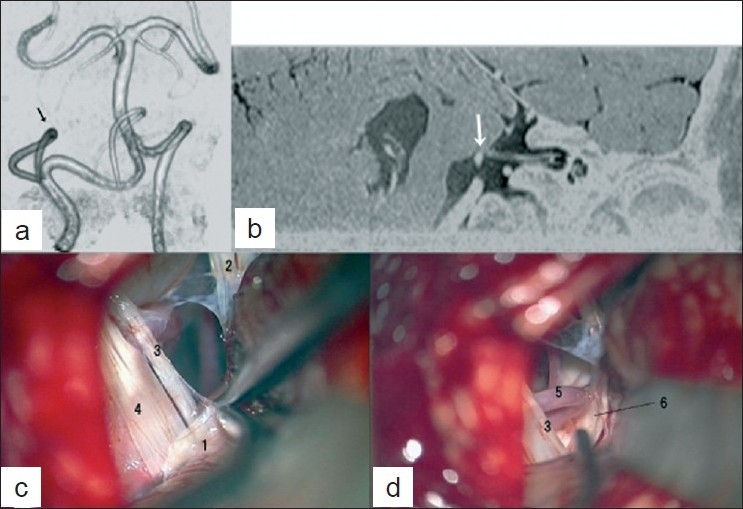
Radiological data and intraoperative findings of a patient with left HFS (Case 1). (a) A preoperative MR angiogram in posterior view showing the left PICA which forms a rostral loop at the high level (black arrow). (b) A preoperative MR image illustrating the offending vessel of the PICA at the root exit zone of the VII nerve (white arrow). (c) An intraoperative view showing that a large rhomboid lip (1) adheres to the IX (3) and X (4) cranial nerves; (2) indicates the VIII cranial nerves. (d) Another intraoperative view after separating the rhomboid lip from the IX and X cranial nerves illustrating clearly visualized root exit zone of the VII nerve (6) and the offending PICA (5)

A 59-year-old female presenting with left-sided HFS for 2 years was admitted to our hospital. Neurological examinations demonstrated typical HFS; it is characterized by twitching tonic spasm and synkinesis of the facial muscles. There were no other symptoms. The preoperative magnetic resonance (MR) angiogram revealed that the left posterior inferior cerebellar artery (PICA) formed a rostral loop at the level of the internal acoustic meatus [Figure 1a]. MR images also revealed that the loop of the PICA compressed the root exit zone of the VII nerve [Figure 1b].

Microvascular decompression of the left VII cranial nerve was performed through the lateral suboccipital approach. Auditory brainstem evoked response (ABR) and facial electromyography were used for intraoperative neurophysiological monitoring. The patient was placed in a lateral park bench position and craniotomy was performed below the posterior end of the incisura mastoidea along the medial boarder of the inferior half of the sigmoid sinus. After dural opening, dissection of the arachnoid membrane around the lower cranial nerves was started. When the inferolateral margin of the cerebellum and the flocculus were gently elevated, a large rhomboid lip was found at the dorsal side of the IX and X cranial nerves [Figure 1c]. The rhomboid lip adhered to the cranial nerves and was meticulously separated from the cranial nerves so that the rhomboid lip and the choroid plexus of the foramen of Luschka could be safely lifted with a spatula. After the procedure, the root exit zone was clearly visualized and the offending artery was observed [Figure 1d]. The offending artery was carefully detached from the root exit zone and a small piece of sponge was placed between the artery and brainstem without manipulation of the VII cranial nerve. The HFS was resolved following the surgery. The postoperative course was uneventful without any new deficits.

### Case 2

**Figure 2:**
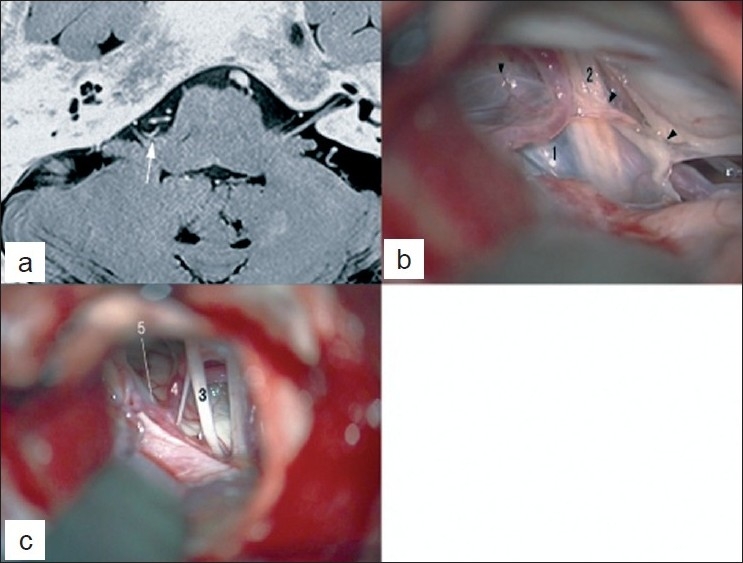
Radiological data and intraoperative findings of a patient with right HFS (Case 2). (a) A preoperative MR image showing the offending vessel of the PICA at the root exit zone of the VII nerve (white arrow). (b) An intraoperative view showing an extremely large rhomboid lip (1 and black arrowhead) which adheres to the X (2) cranial nerve and obstructs the approach to the root exit zone (note that the rhomboid lip is intentionally incised). (c) An intraoperative view after separating the rhomboid lip from the IX (3) and X cranial nerves, illustrating clearly visualized root exit zone of the VII nerve (5) and the offending PICA (4)

A 60-year-old male presenting with right-sided HFS for 15 years was admitted to our hospital. Neurological examinations demonstrated typical HFS. The preoperative MR angiogram revealed that the right PICA formed a rostral loop at the level of the internal acoustic meatus. MR images revealed that the PICA compressed the root exit zone of the VII cranial nerve [Figure 2a].

Microvascular decompression of the right VII cranial nerve was performed through the lateral suboccipital approach with the aid of the same intraoperative monitoring as in Case 1. Intradurally, a large rhomboid lip prevented the visual tract for the root exit zone of the VII cranial nerve and adhered to the dorsal side of the IX and X cranial nerves [Figure 2b]. The surgeon incised the membrane of the rhomboid lip and separated the rhomboid lip from the cranial nerves. After the procedure, the choroid plexus of the foramen of Luschka was safely retracted in a rostral direction with the rhomboid lip, and the root exit zone was clearly visualized [Figure 2c]. The PICA was observed to compress the root exit zone. The PICA was carefully detached from the root exit zone and transposed using the sling retraction method[7] because there was no perforating artery from the PICA. The HFS completely disappeared after the surgery.

## DISCUSSION

### Anatomical relationship among lateral recess, foramen of Luschka, and rhomboid lip

**Figure 3:**
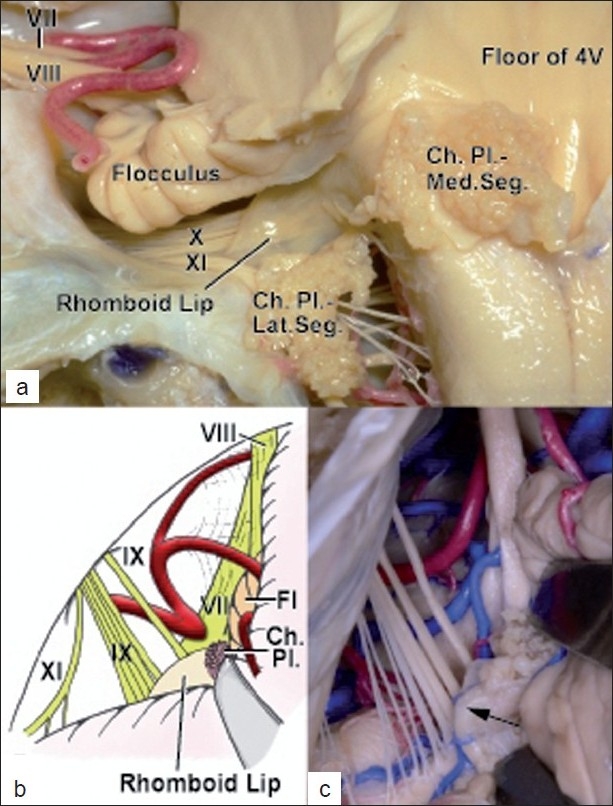
Microsurgical anatomy of the rhomboid lip and the lateral suboccipital infrafloccular approach. (a) Posterior view of the left lateral recess (tela choroidea is reflected inferiorly by cutting tenia). The rhomboid lip is located at the dorsal side of the lower cranial nerves. (b) A schema of intraoperative view of the lateral suboccipital infrafloccular approach in the left side. The tip of the spatula is placed on the choroid plexus protruding from the foramen of Luschka. (c) A cadaveric specimen demonstrating an operative view of the lateral suboccipital infrafloccular approach in the left side. The rhomboid lip (arrow) is identified at the dorsal side of the IX and X cranial nerves, with the choroid plexus protruding from the foramen of Luschka and attaches to the lower cranial nerves. The lateral recess curves slight rostrally and the foramen of Luschka opens to the rostral direction. Ch., choroid; Fl, flocculus; Lat., lateral; Med., medial; Pl., plexus; Seg., segment; 4V, fourth ventricle

The lateral recesses are narrow, curved pouches formed by the union of the roof and the floor of the fourth ventricle. They extend laterally below the cerebellar peduncles and open through the foramina of Luschka into the cerebellopontine angles. The ventral wall of each lateral recess is formed by the floor of the fourth ventricle and the rhomboid lip, which is a sheet-like layer of neural tissue that extends laterally from the floor and unites with the tela choroidea to form a pouch at the outer extremity of the lateral recess [Figure 3a].[6,11] The rostral wall of each lateral recess is formed by the caudal margin of the cerebellar peduncles, and the caudal wall of each lateral recess is formed by the tela choroidea. Thus, proceeding laterally, the rhomboid lip constructs the ventral margin of the foramen of Luschka, and the tela choroidea and accompanying choroid plexus form the dorsal margin.[3,6,11] The origins of the IX and X cranial nerves locate immediately ventral to the foramen of Luschka, and a large rhomboid lip can be identified at the dorsal side of the IX and X cranial nerves, with the choroid plexus protruding from the foramen of Luschka in the lateral suboccipital approach [Figure 3b,c].

### Rhomboid lip as special consideration for hemifacial spasm surgery

The choroid plexus protruding from the foramen of Luschka is an important operative landmark of the root exit zone of the VII cranial nerve during microvascular decompression through the lateral suboccipital approach.[1,4,5,10,12,13] The root exit zone is located medially to the choroid plexus. To reach the root exit zone of the VII cranial nerve without stretching the VIII cranial nerve, the approaches along the inferolateral aspect of the cerebellum are preferred.[8,10] We have adopted a variation of such approaches, the *lateral suboccipital infrafloccular approach*, in which the flocculus is gently retracted in a caudorostral direction perpendicular to the VIII cranial nerve [Figure 3b,c].[1,4,5] This approach facilitates the observation of the root exit zone of the VII nerve with minimal retraction. The tip of the spatula ordinarily must be placed on the choroid plexus to expose the root exit zone of the VII cranial nerves through any of the approaches.[1,4,5,10,12,13] If the rhomboid lip is large and adheres to the IX and X cranial nerves, then the retraction can increase the risk of postoperative hoarseness or dysphagia because of the extension of the IX and X cranial nerves. Lower cranial nerve (IX–XI) deficit was seen in 2.8% of the patients receiving microvascular decompression for HFS.[2] We speculate that retracting the choroid plexus or the adjacent area without noticing the rhomboid lip adhering to the IX and X cranial nerves is one of the possible causes of postoperative lower cranial nerve palsy. The surgeon should meticulously separate the large rhomboid lip from the IX and X cranial nerves before retracting this area and exposing the root exit zone of the VII nerve. When the rhomboid lip is extremely large and completely obstructs the approach to the root exit zone, incising the membrane of the rhomboid lip can facilitate the separation of the rhomboid lip from the lower cranial nerves, as mentioned in Case 2, because breaking the rhomboid lip results in no neurological deficit. ABR and electromyographic monitoring of the lower cranial nerves[9] can also be used for avoiding the cranial nerve damages in such a situation, although we do not routinely use the lower cranial nerve monitoring for HFS surgery.

To our knowledge, adhesion of the large rhomboid lip to the lower cranial nerves has not been mentioned in the literature on microvasular decompression although the situation does not seem uncommon. One of the possible reasons is that surgeons can mistake the rhomboid lip for part of the arachnoid membrane even when the rhomboid lip is large. The rhomboid lip is a slightly thicker and tougher structure than the arachnoid membrane, and usually does not seem as transparent as the arachnoid membrane. It may still be difficult to distinguish between the arachnoid membrane and the rhomboid lip, especially without anatomical knowledge of the rhomboid lip. Another possible reason for the large rhomboid lip, which has not been mentioned, is that the identification of the rhomboid lip might be difficult especially through the lateral route to the root exit zone of the VII cranial nerve. The infrafloccular approach seems suitable for identifying the large rhomboid lip from the anatomical aspect [Figure 3b,c], and the approach can be useful for avoiding unexpected hoarseness or dysphagia after microvascular decompression for HFS.

This report sheds light on the clinical importance of the rhomboid lip during microvascular decompression. The anatomical knowledge of the rhomboid lip and the adjacent area may facilitate safer exposure of the root exit zone of the VII cranial nerve. Further studies on the anatomical variation of the rhomboid lip in its size or distance to the cranial nerves are needed to reinforce the importance of the structure for microvascular decompression.
